# Ultrastructural analysis of bone formation around dental implants in nondiabetic rats, severe diabetics not controlled and controlled with insulin^1^

**DOI:** 10.1590/ACB351101

**Published:** 2020-12-18

**Authors:** Augusto César Rodrigues de Souza, Bruna Aliotto Nalin Tedesco, Pedro Luiz Toledo de Arruda Lourenção, Simone Antunes Terra, Carlos dos Reis Pereira de Araújo, César Tadeu Spadella, Erika Veruska Paiva Ortolan

**Affiliations:** I Fellow Master degree, Postgraduate Program in General Basis of Surgery, Botucatu Medical School, Universidade Estadual Paulista (UNESP), Botucatu-SP, Brazil. Conception and design of the study; acquisition, analysis and interpretation of data; technical procedures; histopathological examinations.; II Fellow Master degree, Postgraduate Program in General Basis of Surgery, Botucatu Medical School, UNESP, Botucatu-SP, Brazil. Conception and design of the study, manuscript preparation and writing.; III Associate Professor, Department of Surgery and Orthopedics, Botucatu Medical School, UNESP, Botucatu-SP, Brazil. Statistics analysis, analysis and interpretation of data, technical procedures, critical revision.; IV Assistant Professor, Department of Pathology, Botucatu Medical School, UNESP, Botucatu-SP, Brazil. Technical procedures, histopathological examinations.; V Assistant Professor, Faculty of Dentistry, Universidade de São Paulo (USP), Bauru-SP, Brazil. Substantive scientific and intellectual contributions to the study, conception and design, critical revision.; VI Full Professor, Department of Surgery and Orthopedics, Botucatu Medical School, UNESP, Botucatu-SP, Brazil. Substantive scientific and intellectual contributions to the study, conception and design, critical revision, final approval.; VII Associate Professor, Department of Surgery and Orthopedics, Botucatu Medical School, UNESP, Botucatu-SP, Brazil. Substantive scientific and intellectual contributions to the study, conception and design, critical revision, final approval.

**Keywords:** Diabetes Mellitus, Dental Implants, Insulin, Osseointegration, Rats

## Abstract

**Purpose:**

To evaluate bone formation through ultrastructural analysis around titanium implants in severe alloxanic uncontrolled diabetic rats, and controlled with insulin, in comparison with nondiabetic rats.

**Methods:**

Thirty-six male Wistar rats, weighing between 200 and 300 g, divided into three experimental groups: normal control group (G1), a diabetic group without treatment (G2), and a diabetic group treated with insulin (G3). The animals received titanium implants in the right femur, and osseointegration was evaluated at 7, 14, and 21 days after surgery, through ultrastructural analysis using scanning electron microscopy.

**Results:**

The ultrastructural analysis showed a dense bone structure in the G1, few empty spaces and a small number of proteoglycans; G2 presented bone matrix with a loose aspect, irregular arrangement, thin trabeculae, empty spaces and a large number of proteoglycans; G3 obtained similar results to G1, however with a higher number of proteoglycans.

**Conclusion:**

Severe diabetes caused ultrastructural changes in bone formation, and insulin therapy allowed an improvement in osseointegration, but it was not possible to reach the results obtained in the control group.

## Introduction

Patients with diabetes mellitus (DM) have an increased risk for the development of periodontal disease^1^,^2^. The severity of periodontal disease leads to alveolar bone loss and consequent loss of the dental element^3^,^4^. Considering the high prevalence of DM and the continuous teeth loss by these individuals, treatments using osseointegrated implants tend to be very useful in these patients.

It is known that the success of the osseointegration implant process depends on several factors. Many studies have been carried out to evaluate the best material and the best treatment of the contact surface of the implant^5^-^7^. In contrast, few studies 8 have been conducted to assess how the clinical condition of the host affects graft osseointegration.

Experimental studies show that bone-implant contact is decreased in diabetic rats compared to control animals, suggesting that the osseointegration process is affected by DM^9^,^10^. On the other hand, insulin administration in diabetic rats maintains bone contact with dental implants, demonstrating that these changes can be reversed through metabolic control with insulin^11^.

The process of osseointegration of the implant in diabetic and nondiabetic patients still needs to be clarified. The present study brings new information through the ultrastructural analysis of bone formation around the implant in uncontrolled alloxanic diabetic rats, diabetic rats controlled with insulin, and nondiabetic rats.

## Methods

This study was approved by the Animal Experimentation Ethics Committee of the Botucatu Medical School - UNESP, under protocol No. 700.

Thirty-six adult male allogenic Wistar rats were used, with approximately 3 months of age and weighing between 200 and 300 g, divided in three experimental groups of 12 animals each: control group (G1), constituted of healthy, nondiabetics rats; diabetic group (G2), consisting of severe diabetic rats induced by alloxan, without treatment; and insulin group (G3), consisting of severe diabetic rats induced by alloxan, treated with insulin.

### Diabetes induction

Diabetes was induced by the administration of 2% alloxan (5,6 dioxyuracil monohydrate – Sigma Co, USA), intravenously in a single dose of 42 mg/kg of body weight, using one of the animal’s tail veins. The animals were followed up for 14 days, being placed in metabolic cages on the 7th and 14th days, aiming for the selection of serious diabetic animals. The animals that presented postinduction blood glucose values above 200 mg/dL and glycosuria greater than or equal to 3+, measured on reagent strips on the 7th and 14th days after induction with alloxan, were considered severe diabetics. Three hundred and sixty-four animals were used in this study, 340 underwent intravenous alloxan injection, and 24 comprised the control group. Of the animals submitted to an alloxan injection, 163 did not become diabetic or did it mildly or moderately, and, for this reason, they were excluded from the study, and 43 died shortly after the injection. The remaining 134 animals became severely diabetic, 20 died during the experiment due to complications from diabetes and 16 due to insulin treatment and prolonged fasting. The group of animals that received insulin had their glycemia normalized in 7 days after the beginning of treatment, through daily administration of human insulin Novolin N, 100 UI/mL (Novo Nordisk A/S, Bagsvaerd – Denmark), in a single dose, via subcutaneous.

Ninety-eight diabetic rats underwent surgery. Of these, 72 remained alive in their respective groups until the time of sacrifice. In the control group, 24 animals were submitted to surgery, with no deaths. Thirty-six animals were randomly selected for analysis by electron microscopy, with the rest destined for other studies.

### Groups

The animals were submitted to an adaptation period of 7 days in polyurethane boxes. After the adaptation period, the animals were initially randomized into two experimental groups (first randomization): In the first group, animals that remained as control (normal nondiabetic group), and in the second, animals that were submitted to experimental diabetes induction with alloxan (diabetic group). After the induction of diabetes, the animals were followed up for 14 days, when they were placed in metabolic cages on the 7th and 14th days after the alloxan injection, aiming at the selection of severe diabetic animals, according to previously established clinical and laboratory criteria.

In the 15th day after induction, diabetic animals were again randomized (second randomization) for the selection of animals that would not receive insulin treatment (G2) and those that would (G3).

**Figure 1:**
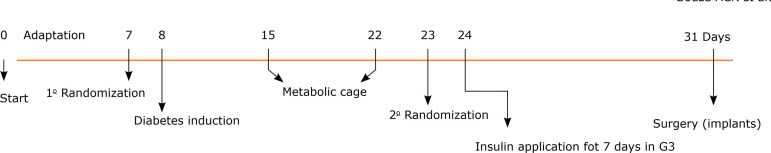
Sequence of procedures perfomed with the animals

The group of animals that received insulin treatment had their blood glucose normalized in 7 days. After glycemia normalization, all animals in the three groups received the implants (Fig. 1).

### Anesthesia

Surgical procedures, including those used for animal sacrifices, were performed using xylazine (0.5 mL/kg of weight) and ketamine (1 mL/kg of weight) administered subcutaneously.

### Implants

**Figure 2:**
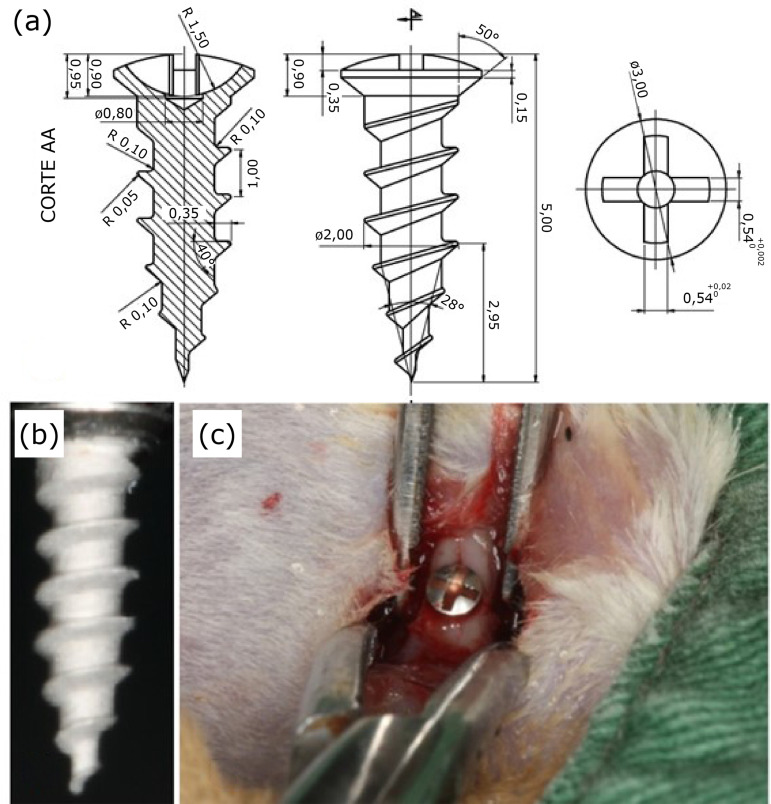
**(a, b)** Design and completion of the implant developed by the engineering sector, **(c)** Implant installed in the animal's femur.

All animals underwent surgery to place one titanium implant in the right femur with measurements of 1.5 mm in diameter and 6 mm in length (Fig. 2 a,b), with NeoPoros surface treatment (Neodent Implantes Osseointegráveis, Curitiba-PR, Brazil), developed especially for this research.

The right lower limb was incised by planes until reaching the musculature that was dissected for exposure of the femur. Bone drilling was performed with the aid of a counter-angle for installing implants with a torque of 20:1 (Antoghir, Injecta, Brazil), coupled to an electric motor (Tecdrill, São Paulo-SP, Brazil) at 800 rpm and abundant irrigation with 0.9% saline solution. The sequence of drills used for drilling the femur was: spear drill, 1.1 mm drill, and 1.3 mm drill. The perforations were 6 mm deep. The fixation of the implants was performed screwing with a manual key until the implant head completely touches the bone cortex. All implants, at the end of this procedure, were in good stability and without signs of mobility (Fig. 2c). After implant placement, a suture in planes was performed with 5-0 nylon.

After the end of the surgical procedure, the rats were housed in individual boxes and placed in a warm environment until complete anesthetic recovery. Approximately 6 h later, water and diet were offered. Antibiotic therapy was performed in all animals by adding tetracycline to the drinking water, at a dose of 50 mg/kg of body weight per day, for 5 days after surgery. During the postoperative period, paracetamol, 10 mg/kg, was administered as an analgesic, in drops diluted in drinking water, for two consecutive days, changed daily.

### Evaluated parameters

Parameters related to osseointegration were analyzed through scanning electron microscopy with a qualitative evaluation of the formation of bone tissue in contact with the surface of the implants.

### Evaluation moments

The osseointegration evaluation moments were: 7 (M7), 14 (M14), and 21 (M21) days after the surgery. Four animals from each group were randomly selected and subsequently sacrificed by cardiac puncture after anesthesia, in each phase of the study, and then the right femur containing the implant was removed.

### Scanning electron microscopy analysis

The samples were fixed in 5% glutaraldehyde in phosphate buffer, pH 7.3 for a period of 7 to 15 days, washed in distilled water (10 times of 10 min each), fixed in 0.5% osmium tetroxide in distilled water for 30 min, dehydrated in an increasing series of ethanol (7.5 – 100%) and dried in a Balzers CPD-020 critical point device, using liquid carbon dioxide.

The analysis of the samples was performed in a scanning electron microscope QUANTA 200 from Fei Company, under voltage of 15 kV, in magnifications of 90 to 3000 times, whose images were digitalized by computer (Pentium-Pro processor, Windows NT system). This analysis observed the characteristics of the newly formed peri-implant bone and bone-implant integration.

## Results

### Ultrastructural analysis with scanning electron microscopy

**Figure 3:**
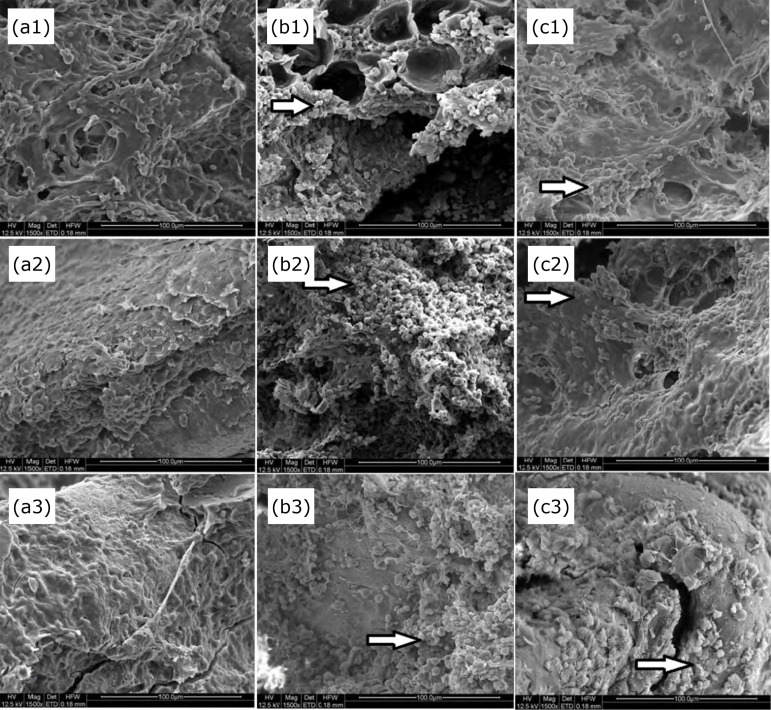
Micrographs of peri-implant bone tissue seen in scanning electron microscopy. **(a)** Control group, **(b)** Diabetic group, **(c)** Insulin group, analyzed at 7, 14 and 21 days after surgery, respectively (1, 2, 3). White arrows indicate proteoglycans (×1500 magnification).

**Figure 4:**
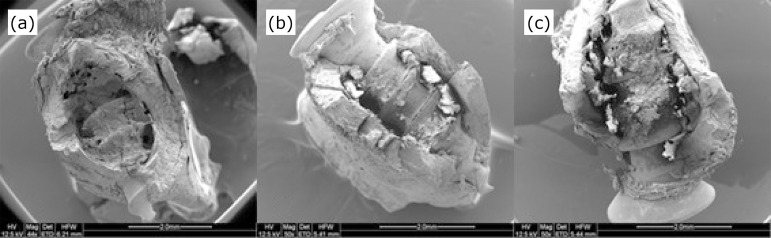
Micrographs of bone tissue, on the 21st postoperative day, on scanning electron microscopy, **(a)** showing animals from the control, **(b)** diabetic and **(c)** insulin groups (50× magnification).

In the higher magnification, differences in the composition of the matrix were evident. In M7, G1 already had a dense bone structure, with few empty spaces and a small number of proteoglycans. G3 was similar to G1, but with a higher number of proteoglycans. The diabetic group (G2), on the other hand, presented loose-looking bone matrix, irregular arrangement, thin trabeculae, more empty spaces, and the presence of a large number of proteoglycans. On the 14th postoperative day (M14), the bone matrix became even denser and defined in G1 and G3, with the maintenance of the presence of proteoglycans in the latter. G2 maintained the aspect of M7, with little evolution of the bone matrix, and with a large number of proteoglycans. In M21, the bone tissue was already fully formed and dense in G1. In G2, the presence of proteoglycans was still remarkable, and thin trabeculae were observed. Although G3 presented bone tissue that was formed and dense, there was still a small number of proteoglycans (Fig. 3). In a small magnification (×50) that allowed a panoramic view of the pieces, in the last analyzed moment, with 21 days of postoperative, it was observed that bone tissue in G1 could be seen in all sides of the implant and in its surface, in intimate contact with the grooves of that. G2 presented space between the implant and the newly formed bone tissue, in addition to having practically no bone material on its surface, revealing failure in osseointegration. The insulin group showed intermediate results between these two groups, with smaller spaces between the bone tissue and the implant and the presence of tissue also on its surface (Fig. 4).

## Discussion

The bone matrix is composed of an organic part (collagen fibrils, elastic fibers, and proteoglycans) and an inorganic part whose composition is given by phosphate and calcium ions forming hydroxyapatite crystals, which offer great mechanical resistance. The organic part is composed mainly of type I collagen. In a small amount, proteoglycans are found in the extracellular matrix, which are main proteins, to which glycosaminoglycan chains are attached^12^. Proteoglycans have different structures and functions, participating in cell adhesion phenomena^13^-^15^. Among the existing proteoglycans, the small proteoglycans rich in leucine can be mentioned. They are considered “small” because they have a central protein that varies from 40 to 60 kDa^16^. Bioglycan is an example of a small proteoglycan expressed in bones, whose deficiency leads to an abnormality of collagen fibrils. Of the 12 small proteoglycans rich in leucine known, 9 are found in bone tissue and appear to have an indirect role in the assembly and function of collagen^17^,^18^. Several authors suggest that the diameter of collagen fibers may be related to the concentration of proteoglycans, and the greater the number of proteoglycans, the smaller the diameter of collagen fibrils^19^-^21^.

It is known, through analysis of transmission electron microscopy, that there is a layer of proteoglycans separating the collagen from the implant surface in the bone-implant contact. Through the aid of electron microscopy, it is possible to verify the production of a thin film (200 to 300 Å) of proteoglycans at the bone-titanium interface. It is adhered to the titanium face and interposed between the screw initially stripped and the alveolar bone. In the outermost portion of the proteoglycan film, close to the alveolar bone, collagen fibrils are randomly arranged^22^.

In the study of osseointegration of dental implants, the researches using scanning electron microscopy, in the vast majority of times, aimed the evaluation of osseointegration on the surfaces of the different types of implants, without considering the most important factor for the adequate integration, which are the characteristics of the recipients of these implants.

Scanning electron microscopy has already been used in studies of the effects of diabetes, with or without surgical interventions, in retina, bones, intestines and kidneys of rats and monkeys, among other organs^23^-^26^. A study that analyzed the bone tissue formed around Kirschner wires in femurs of genetically modified diabetic animals, without treatment and with insulin treatment, through scanning electron microscopy, with 7, 14, 24 and 42 days after the operation, concluded that the initial stages of bone healing are the most affected by diabetes^26^. Experimental studies of the effects of diabetes on osseointegration of dental implants using scanning electron microscopy are valuable to study the bone formation, providing the analysis and description of samples in a larger size, allowing a global view of the arrangement structure of the bone matrix.

In the present study, G1 showed intimate contact between the newly formed bone and the implant surface, which did not occur in G2, where spaces were observed in these same areas. In G3, smaller spaces were noticed. Bone tissue showed a dense structure in G1, with few empty spaces, as well as in G3. The same did not occur in G2, which presented a loose-looking bone matrix, irregular arrangement, thin trabeculae, and more empty spaces. Proteoglycans were present in high amounts in G2, discrete amounts in G1, and moderate amounts in G3.

The massive presence of proteoglycans in diabetic animals may be the mechanism responsible for the fact that the matrix has a looser aspect and is composed of thin trabeculae, with large empty spaces. This immaturity of the matrix in diabetic rats without treatment shows that the bone tissue formed around the implants does not have the same quality when compared to the control group. The results of bone tissue formation in diabetic animals treated with insulin were similar to the animals in the control group, suggesting a higher quality when compared to the diabetic group. However, the presence of proteoglycans in this group suggests a delay in the organization and maturation of the matrix.

## Conclusions

In the present study, experimental diabetes caused ultrastructural changes in the osseointegration of implants in the femur of diabetic rats. Insulin therapy allowed an improvement in the osseointegration of the implants, although it was not possible to reach the results obtained in the control group.
